# Case in Which a Biliary Calculus of Unusual Size Was Safely Passed

**Published:** 1844-04-20

**Authors:** James Couper

**Affiliations:** Newcastle, Del.


### CASE IN WHICH A BILIARY CALCULUS OF UNUSUAL SIZE WAS SAFELY PASSED.

By James Couper, M. D, of Newcastle, Del.

Miss —— had her first attack of gall-stones on the 11th of October, 1841. It produced jaundice, and lasted for nearly a month. Her sufferings were occasionally very severe, but terminated on the expulsion of three or four small calculi. From the fall of 1841 to the month of December 1843, her general health was very good. On the 18th of that month she had another attack, which lasted until the 22d. The agony she endured for two days and nights before the stone left the gall duct was beyond description. Her life was evidently in great danger. On the 22d, the violent pain ceased rather suddenly, and on the next day she passed a biliary calculus of very unusual dimensions. When first discovered, it was covered with thick mucus. Its shape was globular. When cleared of mucus by washing, it measured nine-tenths of an inch in the longer diameter, and eight-tenths of an inch in the shorter. Its weight was not ascertained until after it had been kept for two weeks in a dry room. It then weighed seventy grains, apothecaries' weight. The colour of it was a dark yellow, and there seemed to be several distinct nuclei, which were of a darker hue than the rest of the stone. It is remarkable that no jaundice was produced on this occasion. A moderate degree of soreness over the region of the gall-bladder for a day or two was all the suffering that followed this severe attack.

Little need be said of the treatment. My object was to relax the system by free bleeding, to allay the pain by large doses of the sulphate of morphia, and to keep up the action of the bowels. This last point I accomplished by means of Dr. O'Bierne's invaluable rectum tube. Without it, I must have failed in this part of my plan, on account of the excessive irritability of the stomach and rectum.

By the politeness of our esteemed correspondent we have been permitted "to see and to handle" the gall-stone described in the above communication. It is fully of the size which he mentions, notwithstanding the loss of some particles from its surface and the contraction from drying. It is astonishing indeed that such masses can pass through the slender gall-ducts without more serious consequences than commonly happen. Morgagni, to whose great work our correspondent has referred us, (*De Causis et Sedibus Morborum*) mentions some remarkable cases of the kind, among others that of "the mother-in-law of the celebrated Van Swieten, who was liable to periodical paroxysms of jaundice." "At the end of two days, after very severe and excruciating pains in the seat of the duodenum itself," she "discharged one which equalled the joint of a man's thumb, and shortly after, two more of the same size, which bore marks of having been attached to the first. "And yet the great bulk of this calculus had not prevented it from struggling through the narrow passages of the ducts." "Nor is it to be wondered at," says the same author, "for although the ductus choledochus is narrow, although the cystic is still more narrow, and the passage of it impeded by valves, they are nevertheless membranous, and, for that reason, can bear almost incredible dilatation."

